# Effects of minocycline on the expression of NGF and HSP70 and its neuroprotection role following intracerebral hemorrhage in rats

**DOI:** 10.1016/S1674-8301(11)60040-7

**Published:** 2011-07

**Authors:** Jingnan Pu, Wei Shi, Zizhang Wang, Ruizhi Wang, Zhenyu Guo, Chongxiao Liu, Jianjun Sun, Ligui Gao, Ren Zhou

**Affiliations:** Department of Neurosurgery, the Second Affiliated Hospital of Medical College of Xi'an Jiaotong University, Xi'an, Shaanxi 710004, China.

**Keywords:** minocycline, intracerebral hemorrhage, nerve growth factor, heat shock protein 70

## Abstract

The present study was aimed to investigate the effects of minocycline (MC) on the expression of nerve growth factor (NGF) and heat shock protein 70 (HSP70) following intracerebral hemorrhage (ICH) in rats, and explore the neuroprotective function of MC. Seventy-eight male SD rats were randomly assigned to three groups: the ICH control group (*n* = 36), ICH intervention group (*n* = 36) and sham operation group (*n* = 6). The ICH control group and ICH intervention group were subdivided into 6 subgroups at 1, 2, 4, 5, 7 and 14 d after ICH with 6 rats in each subgroup. Type IV collagenase was injected into the basal nuclei to establish the ICH model. All rats showed symptoms of the nervous system after the model was established, and the sympotsm in the ICH control group were more serious than the ICH intervention group. The number of NGF-positive cells and HSP70-positive cells in the ICH intervention group was higher than that of the ICH control group. MC administration by intraperitoneal injection can increase the expression of NGF and HSP70. MC may inhibit the activation of microglia, the inflammatory reaction and factors, matrix metalloproteinases and apoptosis, thus protecting neurons. The change of the expression of NGF and HSP70 may be involved in the pathway of neuroprotection by MC.

## INTRODUCTION

Intracerebral hemorrhage (ICH) is typically caused by spontaneous rupture of blood vessels. It is an important public health problem with a high incidence and high mortality and exerts a heavy economic and social burden worldwide. ICH represents 10%-30% of all strokes. Mortality after ICH is higher than that after ischemic stroke, and the prognosis is worse; moreover, there is no effective and reliable treatment for ICH at present[1] and research on ICH is still actively pursued worldwide.

During ICH, blood rapidly enters the brain parenchyma, which may further disrupt the blood brain barrier (BBB), impair delivery of oxygen and glucose to cells and result in secondary bleeding. Brain edema begins within hours, and can last for weeks, which contribute to neurological deterioration by increasing intracranial pressure, even causing a shift in brain structures[2]. The primary injury after ICH is physical destruction that compresses the surrounding structures and increases intracranial pressure. The secondary injury is neurological deterioration including hematoma expansion, edema, inflammation and neuron death in the brain parenchyma surrounding the hematoma[3]. Therefore reducing the secondary injury and rescuing neurons following ICH remains an attractive therapeutic goal. The inflammatory response following ICH is characterized by activation of microglia and astrocytes and production of several molecules, including reactive oxygen species, cytokines and matrix metalloproteinases, which can disrupt the blood brain barrier (BBB). Accordingly, there are reports about reducing cell death and improving behavioral outcomes by anti-inflammation, anti-apoptosis and free radical trapping.

Minocycline (MC), a semi-synthetic tetracycline derivative, has high oral bioavailability, and superior BBB penetration and is well tolerated by humans, where it has been used for decades to treat bacterial infections[4]. MC has shown promise as a neuroprotectant in animal models of several acute and chronic neurological disorders including traumatic head injury, spinal cord injury, Parkinson's disease, Huntington's disease, ischemic stroke and ICH, and its anti-inflammatory, anti-apoptotic and antioxidant properties are thought to underlie its neuroprotective effects[5]–[8].

Nerve growth factor (NGF) and heat shock protein 70 (HSP70) play important roles in brain injury[9]–[12], but few studies on the interaction of MC with NGF and HSP70 have been published in the literature. For further research on the protective effect of MC on neuron after ICH, the present study established a rat model of ICH induced by type IV collagenase to observe whether MC promoted the expression of NGF and HSP70 around the hematoma following ICH at different time points, and exerted therapeutic effect. Thus, it is helpful to illuminate the mechanism of secondary brain injury after ICH, and provide a theoretical basis of clinical therapy and prognosis and new treatment ideas.

## MATERIALS AND METHODS

### Materials

A total of 78 healthy male adult Sprague Dawley rats, weighing 200-250 g, were purchased from the Animal Experiment Center of Xi'an Jiaotong University Medical School [No. SCXK(Shaan)2007-001]. The rats were allowed free access to food and water in a quiet environment at a constant temperature of 20°C-25°C. The experimental protocols were approved by the local institutional review boad and performed in accordance with the Guidelines for Care and Use of Laboratory Animals formulated by the Ministry of Science and Technology of China[13].

### Animal grouping

Seventy-eight rats were randomly assigned to 3 groups: 1) the ICH control group (*n* = 36) was randomly subdivided into 6 subgroups at d 1, 2, 4, 5, 7 and 14 after ICH, with 6 rats in each subgroup; 2) the ICH intervention group (*n* = 36) was randomly subdivided into 6 subgroups at d 1, 2, 4, 5, 7 and 14 after ICH, with 6 rats in each subgroup; 3) the sham operation group (*n* = 6) served as control at d 4 after ICH.

### Establishment of the ICH model

Type IV collagenase was used to establish the ICH model according to the previous method[14]. The rats were anesthetized intraperitoneally with 2% chloral hydrate (350 mg/kg) and then positioned prone on a stereotaxic frame. A midline scalp incision was made, and a hole was drilled in the right skull (1 mm anterior to the bregma, and 3 mm lateral to the midline), and then 1 µL mixture (0.2 U type IV collagenase+2 U heparin+saline) was stereotaxically injected into the right caudate nucleus 5 mm below the surface of the drilled hole in the skull within 5 min, and the needle was left in place for another 5 min. The syringe was then removed slowly. The burr hole in the skull was sealed with bone wax, and the scalp was then sutured. The sham operation group was performed with needle insertion only. At 6 h after ICH was established, MC (Sigma-Aldrich, St. Louis, MO, USA) was intraperitoneally injected at 45 mg/kg, followed by 22.5 mg/kg every 12 h until the rats were sacrificed in the ICH intervention group. The ICH control group and sham operation group were treated with normal saline of the same volume.

### Determination of relevant indicators

The rats were overdosed with 2% chloral hydrate at different time points after ICH. Thoracotomy was performed quickly, and the heart was exposed immediately. Aortic cannulation was performed via the left ventricle. An incision was made at the right atrium, and 100 mL of 4% paraformaldehyde was perfused rapidly. The rats were decapitated after perfusion. Fixed brain was coronally cut into slices with the microsyringe needle tract as the center, approximately 5 mm each. These brain slices were dehydrated for 2-3 d and then sections (20 µm) were cut for immunohistochemical staining.

The neurological deficits in rats were evaluated by Longa FZ: level 0: no signs, score 1; level 1: unable to fully straighten the front legs, score 2; level 2: hemiplegia and rear collision, score 3; level 3: unable to stand or roll, score 4; level 4: no spontaneous activity and disturbance of consciousness, score 5.

### Detecting NGF-positive cells and HSP70-positive cells

Immunohistochemistry DAB method (according to the kit instruction purchased from Boster, Wuhan, China) was used to detect NGF-positive cells and HSP70-positive cells, which were stained as brown-yellow. The sections (20 µm) were incubated with 50 µL H_2_O_2_ at room temperature for 10 min followed by blocking at room temperature for 60 min and were then incubated with rabbit anti-rat NGF antibody (Boster), or rabbit anti-rat HSP70 antibody (Boster) at 4°C overnight. Biotin labeled goat anti-rabbit IgG (Boster) was added at room temperature for 90 min and SABC for 20 min. The sections were colored in DAB, dehydrated, cleared, and mounted with neutral gum[15]. NGF-positive cells and HSP70-positive cells were quantified at 5 visual fields around the hematoma cavity by 400× light microscope (Olympus, Tokyo, Japan).

### Statistical analysis

Quantitative data were expressed as mean±SD. Data analysis and statistics were processed by SPSS 11.5 (Chicago, IL, USA). Statistical significance was verified by two-factor randomized block design analysis of variance. Significance was accepted at *P* < 0.05.

## RESULTS

### The score of nervous system function

No obvious neurological deficits were encountered in the sham operation group from consciousness to death. The ICH control group and ICH intervention group exhibited neurological deficits of different degrees, including loss of energy, impaired response, hemiplegia, rear collision and crawling difficulty. Compared with the ICH control group, the score of nervous system function of the ICH intervention group showed statistically significant difference at different time points (*P* < 0.05). Compared with the sham operation group, the scores of nervous system function in the ICH control and ICH intervention group were significantly different (*P* < 0.05, ***Table 1***).

**Table 1:** The score of nervous system function

Group	1 d	2 d	4 d	5 d	7 d	14 d
ICH control group	2.83±0.37^△^*	2.83±0.41^△^*	2.33±0.82^△^*	2.17±0.98^△^*	2.33±1.03^△^*	1.17±0.37^△^*
ICH intervention group	1.67±0.47^△^	1.83±0.98^△^	1.17±0.41^△^	1.17±0.41^△^	1.17±0.61^△^	0.67±0.47
Sham operation group			0.50±0.10			

^△^Compared with the sham operation group, *P* < 0.05; *Compared with the intracerebral hemorrhage (ICH) intervention group, *P* < 0.05.

(mean±SD, *n* = 6)

### NGF immunohistochemistry staining

**Fig. 1:**
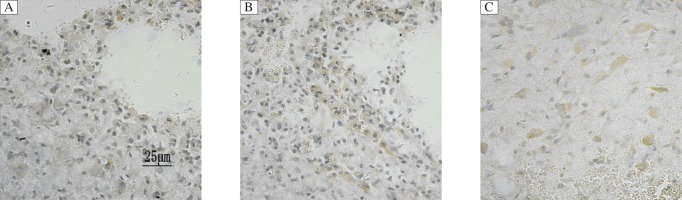
NGF-positive cells in the perihematoma region at d 7 after ICH (immunohistochemical staining, ×400). A: ICH control group. B: ICH intervention group. C: sham operation group. A large number of NGF-positive cells with brown-yellow particles in the cytoplasm and slightly stained nuclei around the hematoma. ICH: intracerebral hemorrhage.

NGF-positive cells were observed around the hematoma, with brown-yellow particles in the cytoplasm and slightly stained nuclei. Little NGF expression was detected in the brain tissue of normal rats. A large number of NGF-positive cells were detected around the hematoma, increased with ICH time, and peaked at 7 days in the ICH control group and ICH intervention group. The number of NGF-positive cells in the ICH control group was fewer than that in the ICH intervention group. Compared with the ICH control group, the number of NGF-positive cells in the ICH intervention group showed statistically significant difference at different time points (*P* < 0.05). There was a statistically significant difference between the sham operation group and ICH control group, the sham operation group and ICH intervention group (*P* < 0.05, ***Table 2*** and ***Fig. 1***). MC treatment increased the number of NGF-positive cells.

**Table 2:** The number of NGF-positive cells

Group	1d	2 d	4 d	5 d	7 d	14 d
ICH control group	229.7±2.0^△^*	232.1±3.2^△^*	233.8±1.4^△^*	241.9±2.6^△^*	247.4±3.3^△^*	240.8±2.6^△^*
ICH intervention group	265.1±2.6^△^	267.2±2.2^△^	272.7±7.9^△^	282.0±2.1^△^	285.5±2.1^△^	281.5±2.0^△^
Sham operation group			68.8±5.8			

^△^Compared with the sham operation group, *P* < 0.05; *Compared with the intracerebral hemorrhage (ICH) intervention group, *P* < 0.05.

(mean±SD, *n* = 6)

### HSP70 immunohistochemistry staining

**Fig. 2:**
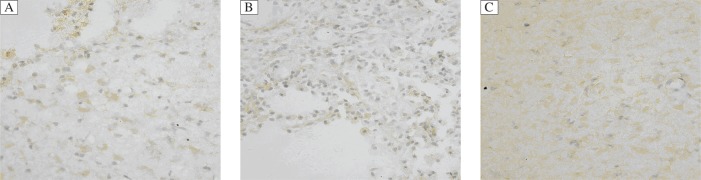
HSP70-positive cells in the perihematoma region at d 7 after ICH (immunohistochemical staining, ×400). A: ICH control group. B: ICH intervention group. C: sham operation group. A large number of HSP70-positive cells with brown-yellow cytoplasm or nuclei in the ischemic penumbra. ICH: intracerebral hemorrhage.

Many HSP70-positive cells with brown-yellow cytoplasm or nuclei were observed around the hematoma. HSP70 was not expressed in the brain tissue of normal rats. HSP70 expression peaked at 24 and 48 h following ICH with higher levels of expression in the ICH intervention group. Compared with the ICH control group, the number of HSP70-positive cells in the ICH intervention group showed statistically significant difference at different time points (*P* < 0.05). There was a statistically significant difference between the sham operation group and ICH control group and ICH intervention group (*P* < 0.05, ***Table 3*** and ***Fig. 2***). MC treatment increased the number of HSP70-positive cells.

**Table 3:** The number of HSP70-positive cells

Group	1 d	2 d	4 d	5 d	7 d	14 d
ICH control group	51.1±1.9^△^*	56.8±0.4^△^*	34.1±1.1^△^*	22.5±0.9^△^*	5.7±0.4^△^*	1.9±0.2^△^*
ICH intervention group	80.8±1.5^△^	89.8±0.9^△^	53.9±0.7^△^	35.8±1.0^△^	7.6±0.3^△^	2.8±0.3^△^
Sham operation group			00.4±1.0			

^△^Compared with the sham operation group, *P* < 0.05; *Compared with intracerebral hemorrhage (ICH) intervention group, *P* < 0.05.

(mean±SD, *n* = 6)

## DISCUSSION

ICH is one of the most lethal types of stroke, and it leaves many survivors disabled. Effective treatments for ICH are still desperately needed at present. Fortunately, the number of experimental ICH studies has increased, and much progress has been made, thus leading to putative therapeutic targets and treatments. Rat models contribute greatly to the elucidation of stroke mechanisms *in vivo*[16]. Infusion of type IV collagenase is the most widely used rat model of ICH. Presumably, the better model should more closely imitate the pathophysiology and functional consequences of ICH in humans while considering practical issues. In this study, we used type IV collagenase injected into the caudate nucleus stereotaxically to establish the ICH model that reflects the clinical pathological process. Type IV collagenase model may cause great structural injury. There are several possible reasons. First, type IV collagenase depending on the dosage and diffusion characteristics damages many blood vessels and results in blood broadly dissecting throughout the parenchyma. More brain cells are exposed to degenerating erythrocytes and probably inflammatory cells, which lead to a greater space occupying effect and edema, and presumably greater neurotoxicity in type IV collagenase model. Second, in the type IV collagenase model, BBB extravasation was significant, especially within the hematoma. The temporal profile of BBB breakdown here is similar to other events, which contribute to damage, including erythrocyte degeneration and edema formation. Third, widespread destruction of the vasculature may cause some ischemic injury after type IV collagenase infusion, at least within the diffuse hematoma, and this may cause severe BBB disruption[17]. The caudate nucleus where ICH frequently occurs is the largest nucleus group and allows localization and injection. We used 1 µL mixture (0.2 U IV collagenase+2 U heparin+saline) to induce hematoma with the same volume and at the same position. The model was stable, with a high success rate and reproducibility confirmed by neurological behaviors, gross observation, and hematoxylin and eosin staining.

NGF as an endogenous neurotrophin makes some contribution to trophic and differentiating activity on neurons of the central and peripheral nervous systems. NGF is important to the growth, maintenance, and survival of certain target neurons[18],[19]. NGF binds to at least two classes of receptors: p75 NTR and TrkA. NGF binds to high-affinity tyrosine kinase receptor, TrkA, which leads to the activation of PI-3K, ras, and PLC signaling pathways. There are a great deal of interests in whether NGF can prevent neurodegenerative diseases, or promote central nervous sysytem repair. As we all know, NGF also binds to the p75NTR, a member of the tumor necrosis factor receptor superfamily. Activation of the p75NTR causes cell death rather than survival. The binding of NGF to TrkA results in receptor phosphorylation, followed by the activation of signaling pathways that enhance cell survival and neuronal differentiation. In contrast, signaling via p75NTR can activate pathways of cell death[20]. Therefore, the final effect of NGF is a balance between cell survival signal derived from the TrkA family and cell death signal from the p75NTR[21]. The effect of NGF may be related not only to a neuroprotective activity against apoptosis, but also to the formation of new neural pathways, as it is known that NGF has the ability to promote neural plasticity and axonal regeneration[22]. There is evidence that NGF circulates throughout the entire body and is important for maintaining homeostasis[23]. The precursor to NGF and pro-NGF may also play important roles due to its apoptotic and neurotrophic properties[24].

HSPs are found in virtually all living organisms, from bacteria to humans in response to environmental challenges, including hyperthermia, excitotoxic exposure, and other stresses[25]. HSP70 plays an important role in the cell's machinery for protein folding, and help to protect cell from stress[26],[27]. HSP70 may be involved in the pathways of inflammation and apoptosis[28]. HSP70 as a sign of irreversible damage to neurons is expressed in the ischemic penumbra during ischemic damage. It is also considered as a sensitive and reliable marker of ischemia[29]. Some investigators think that the overexpression of HSP70 protects neurons from lethal insults. HSP70 overexpressed by genetic manipulations or pharmacological inducers has been reported to exert crucial neuroprotective effects on cerebral focal ischemia, polyglutamine-mediated motor neuron disease, severe heat stress, and other stressful conditions[30]. Others conclude that the overexpression of HSP70 confers protection against cerebral ischemia, results in a smaller lesion volume, and possibly also lessens cellular damage of the lesion within 24 h[31]. Whether HSP70 actually prevents ischemia injury or only delays it remains to be resolved. At the moment the mechanisms by which HSP70 exerts neuroprotective effect against cerebral infarction are not fully understood. Whether HSP70 overexpression could play a clinical role in protecting the brain after injury requires further investigation.

Pharmacologically, MC as a possible therapy has attracted tremendous attention in both acute and chronic brain disorders, including ischemic stroke and ICH. MC in early phase clinical trials is encouraging. As a broad spectrum tetracycline antibiotic, MC is the most lipid-soluble of the tetracycline-class antibiotics, giving it the greatest penetration into the prostate and brain, but also the greatest amount of central nervous system-related side effects. As an anti-inflammatory agent, MC inhibits apoptosis via attenuation of TNF-alpha, downregulating pro-inflammatory cytokine secretion. This effect is mediated by a direct action of MC on activated T cells and microglia, which results in decreased ability of T cells to contact microglia, thus impairing cytokine production in T cell-microglia signal transduction[32]. MC also inhibits microglial activation through blockade of NF-κB nuclear translocation. The present study is eager to provide a better and authentic interpretation of the relationship between inflammation and neuron death following ICH, and further to assess whether MC has neuroprotective effect when treatment is delayed to a time that is relevant to human ICH. MC exerts a series of neuroprotective effects in animal models of brain injury, including anti-inflammatory effect, inhibit microglial activation, reduce production and activity of some matrix metalloproteases, inhibit production of oxygen free radicals and expression of iNOS, and reduce protein tyrosine nitration[3]. Injury to cells around the hematoma may be mediated by a variety of complicated mechanisms, including mechanical injury from clot expansion and retraction, toxicity of hemoglobin and thrombin, and inflammation[33]. It is impossible that any single drug that specifically targets one mechanism will provide an optimal outcome. MC appears to have multiple beneficial functions. Further investigation into MC obviously has a broad prospect since it has been proved to be safe for human administration[34].

Compared with cerebral ischemic stroke, far fewer studies have focused on the relationship between neuron death and inflammation following ICH. There is an expectation and some evidence that cytoprotective treatment will lessen mortality and morbidity. In our study, the results showed that all symptoms of nervous system in rats appeared after the model was established. The ICH control group and ICH intervention group exhibited neurological deficits of different degrees, including loss of energy, impaired response, hemiplegia, rear collision and crawling difficulty. The ICH control group showed greater neurological deficits than the ICH intervention group. Neurological deficit scores showed that MC can promote the recovery of neurological function after ICH. NGF-positive cells and HSP70-positive cells in the ICH intervention group were significantly greater in number than those in the ICH control group after MC administration. MC administration by intraperitoneal injection can increase the expression of NGF and HSP70. The mechanism may be that MC can regulate the expression of TrkA, which binds to NGF with high affinity during cerebral ischemia-reperfusion injury and activates the downstream PI-3K/Akt pathway to inhibit the excessive release of glutamate to reduce brain injury due to Ca^2+^ overload, thereby protecting the brain. MC may enhance hypoxic and ischemic tolerance of the brain, thus playing an important role in neuroprotection. The mechanism may be related with ischemic penumbra, Ca^2+^ overload, free radicals and excitatory amino acids, which can increase the expression of HSP70 and inhibit apoptosis.

Overall, while inflammation and neuroprotection are important outcomes in the experimental setting, successful translation to human therapies will require pre-clinical studies that show improved neurological outcomes. Our study on animal model of the central nervous sysytem demonstrates that MC can increase the expression of NGF and HSP70, both of which play important roles in inhibiting inflammation and apoptosis, protecting neurons, and promoting the recovery of neural function and the healing of brain injury at different stages. With MC administration, the neurological deficits were eased. The expression of NGF and HSP70 in brain tissue following ICH is in accordance with the behavior changes. MC has neuroprotective effect, which relates to the inhibition of microglia, the inflammatory reaction and factors, matrix metalloproteinases and apoptosis. Neuroprotection of MC involves many pathways. The concrete mechanism is still not very clear. We speculate that the change of the expression of NGF and HSP70 may be involved in the pathway.
